# Post-traumatic stress disorder symptoms in COVID-19 survivors: online population survey

**DOI:** 10.1192/bjo.2021.3

**Published:** 2021-02-09

**Authors:** Samuel R. Chamberlain, Jon E. Grant, William Trender, Peter Hellyer, Adam Hampshire

**Affiliations:** Department of Psychiatry, University of Southampton; and Department of Psychiatry, University of Cambridge, UK; Department of Psychiatry, University of Chicago, USA; Department of Brain Sciences, Imperial College London, UK; Department of Brain Sciences, Imperial College London, UK; Department of Brain Sciences, Imperial College London, UK

**Keywords:** Post-traumatic stress disorder, trauma, COVID, coronavirus, COVID-19

## Abstract

This study examined post-traumatic stress disorder (PTSD) symptoms in 13 049 survivors of suspected or confirmed COVID-19, from the UK general population, as a function of severity and hospital admission status. Compared with mild COVID-19, significantly elevated rates of PTSD symptoms were identified in those requiring medical support at home (effect size 0.178 s.d., *P* = 0.0316), those requiring hospital admission without ventilation (effect size 0.234 s.d., *P* = 0.0064) and those requiring hospital admission with ventilator support (effect size 0.454 s.d., *P* < 0.001). Intrusive images were the most prominent elevated symptom. Adequate psychiatric provision for such individuals will be of paramount importance.

Although there has been considerable speculation as to the likely deleterious effects of COVID-19 infection on mental health, some types of mental health symptoms may be especially anticipated to occur in people who have been infected. In a systematic review of the literature examining the mental health impact of COVID-19, only two studies were identified that had evaluated patients with suspected or confirmed SARS-CoV-2 infection, whereas 41 studies had examined indirect effects of the pandemic.^1^ Given that post-traumatic stress disorder (PTSD) is a common consequence of exposure to extremely stressful acute circumstances, high rates of PTSD in COVID-19 survivors have been predicted, especially in cases of severe illness.^2^

Knowledge regarding relationships between viral infections and PTSD in past outbreaks is scant. In previous coronavirus literature (pre-COVID-19), PTSD was examined in just four data studies, with high rates of PTSD being reported.^3^ For the COVID-19 pandemic, initial studies in China have identified high rates of PTSD symptoms in COVID-19 survivors while in-patients and after in-patient discharge.^4,5^ However, these studies did not have reference groups to contextualise the results, such as non-admitted milder cases, drawn from the same recruitment pool. Consequently, they cannot differentiate whether elevated rates of PTSD were due to direct effects of infection and its treatment, or indirect traumatic effects of the pandemic.

The aims of the current study were to examine PTSD symptoms in survivors of suspected or confirmed COVID-19 and to address whether these differed as a function of COVID-19 symptom severity and treatment. We hypothesised that PTSD would be disproportionately elevated in those requiring in-patient admission, especially those requiring ventilation support, compared with those who had mild COVID-19 symptoms that had been managed at home.

## Method

### Overview and participants

Data collection was conducted in May 2020 as part of a broader citizen science study ‘The Great British Intelligence Test’ (GBIT). Articles describing the study were placed on the BBC Two Horizon web page, BBC Home page, BBC News Home page and circulated on mobile news meta-apps. The GBIT study was originally set up to explore intelligence and cognition in the general public. However, with the emergence of the pandemic, we applied for an ethics amendment to include a questionnaire on COVID-19, in addition to our originally planned mental health and cognitive measures. This enabled us to identify people with a self-reported diagnosed history of COVID-19 infection, including those who reported they had received a positive confirmation via a biological test. This had the methodological advantage that none of the advertisements/materials mentioned COVID-19 or PTSD, thereby minimising topic-specific selection bias. Participants completed an online survey accessible via any mainstream device (e.g. smartphones, computers). Inclusion criteria were being aged 16 or older and reporting previously having been ill with COVID-19. The study and its procedures complied with the ethical standards and with the Helsinki Declaration of 1975, as revised in 2008. All procedures were approved by the Imperial College Research Ethics Committee (17IC4009) and all participants provided informed consent.

### Instruments

**Fig. 1:**
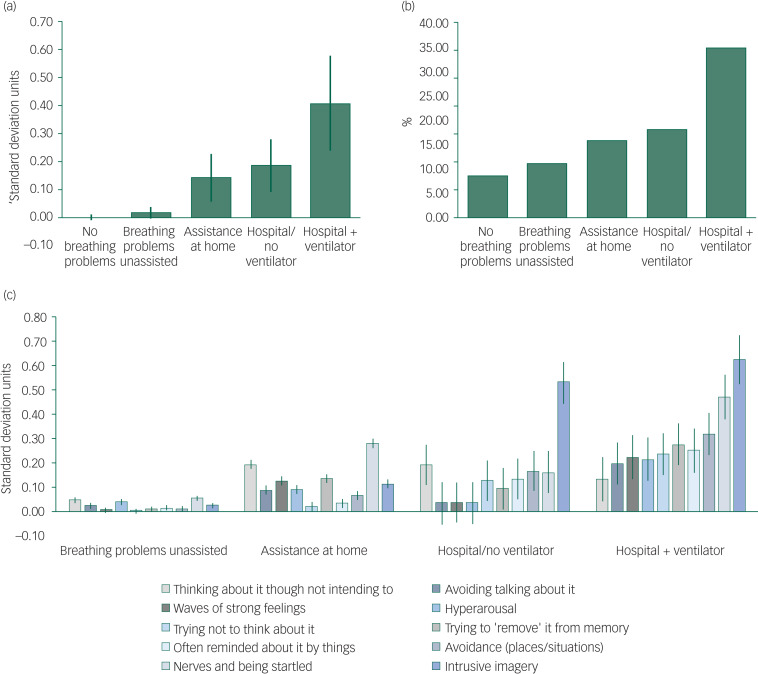
Relationships between COVID-19 severity and subsequent overall and individual-item symptoms of post-traumatic stress disorder (PTSD). (a) Difference in PTSD composite score for groups sorted by respiratory symptoms/treatment relative to the group that reported no breathing difficulties. (b) Percentage of people within each group who endorsed all ten symptoms as applying to them to some degree. (c) Individual-item analyses per group in s.d. units relative to people who reported no breathing difficulties. Composite and item score data are presented after controlling for potential confounders (demographic characteristics, medical/psychiatric history and non-PTSD-specific mood/anxiety symptoms). Error bars show the s.e.m.

Questionnaire items included: demographic characteristics (age, gender, income, educational level, occupational status, ethnic group, country of residence, first language and earnings); details of past COVID-19 infection and medical support received (the questions asked are listed in the supplementary material, available at https://doi.org/10.1192/bjo.2021.3); the Generalized Anxiety Disorder Assessment 7 (GAD-7)^6^ and Patient Health Questionnaire 2 (PHQ-2)^7^ to account for anxiety and depression respectively; reports of pre-existing psychiatric and neurological diagnoses, as well as other common medical conditions associated with COVID-19 susceptibility (see supplementary material for questions); and questions from the Impact of Events Scale Revised (IES-R).^8^ For the IES-R, we selected the ten items most pertinent to the impact of COVID-19, based on consensus among the study team (Fig. 1(c)). This was necessary owing to the need for a short questionnaire. The IES-R is based on DSM-IV criteria but was chosen as it had been deployed in other COVID-19 research when we selected the study measures. Participants were instructed to respond to the IES-R on the basis of their COVID illness (i.e. it was anchored to COVID illness experience, rather than other types of trauma).

### Data analysis

Statistical analyses were conducted in MATLAB R2020a for Windows. The outcome measures of interest were IES-R responses. Linear regression was then applied to factor out effects of demographic characteristics, non-PTSD specific anxiety and depressive symptoms (GAD-7 and PHQ-2) and pre-existing mental, neurological and physical health conditions. Binary response data were coded as 1 for yes and 0 for no. Residuals were entered into analysis of variance models to compare groups first on overall PTSD total score (mean of all questionnaire answers transformed to s.d. units) and then as a function of individual PTSD items (also transformed to have unit deviation). The groups of interest were: no breathing problems, breathing problems unassisted, assistance at home, hospital admission without ventilation and hospital admission with ventilation. We used s.d. units since these are equivalent to Cohen's *D* effect sizes, which makes it easier to interpret findings. For large data-sets, reporting only *P*-values can be misleading – hence both *P* and the equivalent of Cohen's *D* were indicated.

## Results

In total, 13 049 people took part in the study and reported that they had a history of COVID-19 (see supplementary Table 1 for their demographic characteristics). Of these, 9200 (70.5%) reported they had not experienced breathing problems, 3466 (26.6%) reported breathing problems but not requiring medical input, 176 (1.3%) reported they had had breathing problems and had assistance at home, 147 (1.1%) reported hospital admission but without needing a ventilator and 60 (0.5%) reported hospital admission including ventilation. Overall, 361 (2.8%) of these individuals reported that they had received a confirmatory biological test result.

It can be seen from Fig. 1 that overall PTSD symptom scores varied significantly across the groups (*F*(4,13044) = 6.7383, *P* = 2.06E-05). This was due to greater PTSD levels in the groups with more severe respiratory symptoms relative to those who had no respiratory symptoms (no assistance for respiratory symptoms: 0.037 s.d., *P* = 0.1228; assistance at home: 0.178 s.d., *P* = 0.0266; hospital, no ventilator: 0.234 s.d., *P* = 0.0058; hospital with ventilator: 0.454 s.d., *P* = 0.001). However, when analysed to include whether individuals had received a confirmatory COVID-19 biological test as a factor, there was no main effect of confirmatory test and the effect size was of negligible scale (*F*(1,13043) = 1.6139, *P* = 0.2040, estimated 0.073 s.d.), whereas the main effect of respiratory group remained robust (*F*(4,13043) = 5.2954 *P* = 0.0003).

Further analysis of item-level data from the IES-R showed that responses were sensitive to respiratory symptom group (Fig. 1; for statistical details see supplementary Tables 2 and 3). In particular, intrusive PTSD imagery was most common in the groups with higher-severity COVID-19.

## Discussion

This study examined PTSD symptoms in survivors of suspected or confirmed COVID-19 recruited from the general population predominantly in the UK. Our data confirmed the hypothesis that PTSD symptoms were disproportionately elevated in those requiring in-patient admission, especially those requiring ventilation support, compared with those who had mild COVID-19 symptoms that had been managed at home. These group-level differences controlled for relevant demographic characteristics, medical and psychiatric history, as well as background levels of anxiety and depression. Because we recruited from a general population sample, the findings are more likely to be generalisable to the public at large; and risk of topic-specific selection bias is lower since our study advertisement materials did not mention COVID-19 or PTSD.

### Limitations

The study used an abbreviated self-report instrument and examined dimensional PTSD symptoms, rather than identifying formal diagnoses, which would require in-person clinical interviews and confirmation of sustained symptoms over time. Nonetheless, for context, the overall percentage of the sample indicating that at least one symptom of PTSD applied ‘extremely’ to them was 41%; and 35% of previously admitted patients needing ventilator support endorsed all ten symptoms to some degree (Fig. 1). Effect sizes were generally small compared with cases with no respiratory symptoms, albeit the elevated rates of PTSD in those requiring ventilator support was approaching medium effect size (*D* = 0.45) and was especially noteworthy for experiencing intrusive imagery (*D* = 0.63). Relatively small effects could partly relate to the methodology (online data collection) and the statistical control for confounders (which may yield conservative estimates). It should be considered that there is likely to be heterogeneity: even if the averages were of small effect, of course within those samples there would be people who experienced more severe/pronounced PTSD owing to higher vulnerability than others.

Rates of biologically confirmed COVID-19 in our sample were low (2.8%): this likely reflects our sampling period, which was prior to biological tests becoming widely available. Owing to the low rates of biological confirmation, some individuals may have reported a diagnosis of COVID-19 that was in error (e.g. apparent infection due to other causes besides COVID-19).

We assessed PTSD symptoms but did not make formal diagnoses, since this study did not involve a clinical interview. In particular, the screening tool could not determine that the formal definition of ‘trauma’ was met in relation to a person's COVID-19 experiences, nor that PTSD symptoms had persisted for sufficient time to constitute PTSD rather than an adjustment reaction. Similarly, time since infection was not assessed. Many would consider that being diagnosed with a potentially life-changing illness can itself constitute ‘trauma’, although the precise definition of what constitutes ‘trauma’ in PTSD remains debated and has changed from DSM-IV to DSM-5. The instrument we used was based on DSM-IV.

### Clinical and research implications

Collectively, these findings highlight the importance of following up survivors of COVID-19 infection for PTSD. The current cross-sectional data provide impetus to examine PTSD symptoms longitudinally in COVID-19 survivors, to further inform the need for healthcare provision in this setting, and understand the direct and indirect impact of the pandemic.

## Data Availability

The authors will consider reasonable requests for data access from members of academic institutions; requests should be submitted to the corresponding author.

## Supplementary material

For supplementary material accompanying this paper visit https://doi.org/10.1192/bjo.2021.3. click here to view supplementary material
