# Supplementary material for: Post-traumatic stress disorder symptoms in COVID-19 survivors: online population survey
Source: BJPsych Open. 2021 Feb 9;7(2):e47. doi: 10.1192/bjo.2021.3 (PMC7873456; doi:10.1192/bjo.2021.3)
Supplement: Supplementary file 1 [file S205647242100003Xsup001.docx]

Supplementary File – Chamberlain et al., COVID-19 infection and PTSD

Table S1. Demographic characteristics of the sample.

| **Sex** | **Counts** |
| --- | --- |
| Female | 7064 |
| Male | 5915 |
| Other | 70 |
|  |  |
| **Handedness** | **Counts** |
| Ambidextrous | 421 |
| Left handed | 1380 |
| Right handed | 11248 |
|  |  |
| **First language** | **Counts** |
| English | 12071 |
| Other | 978 |
|  |  |
| **Resident** | **Counts** |
| United Kingdom | 12088 |
| other | 961 |
|  |  |
| **Ethnicity** | **Counts** |
| Rom, Sinti or Bedouin | 8 |
| North African | 20 |
| Sub-saharan African or Afro-american | 54 |
| West-central Asian | 54 |
| American Hispanic | 79 |
| East Asian | 157 |
| Unknown | 172 |
| Indian, South Asian or South-East Asian | 408 |
| Mixed ethnicity | 421 |
| White European or North American | 11676 |
|  |  |
| **Education level** | **Counts** |
| 01 No schooling | 38 |
| 02 Primary/Elementary school | 221 |
| 03 Secondary school/High school diploma | 4283 |
| 04 University degree | 7962 |
| 05 PhD | 545 |
|  |  |
| **Occupational status** | **Counts** |
| Disabled/Not applicable/Shielded employment | 161 |
| Homemaker | 445 |
| Retired | 1567 |
| Student | 1151 |
| Unemployed/Looking for work | 466 |
| Unknown | 59 |
| Worker | 9200 |
|  |  |
| **Earnings** | **Counts** |
| Not working | 3849 |
| prefer not to say | 286 |
| £0-10K | 120 |
| £10-20K | 1343 |
| £20-30K | 1876 |
| £30-40K | 1740 |
| £40-50K | 1264 |
| £50-60K | 773 |
| £60-70K | 414 |
| £70-80K | 318 |
| £80-90K | 202 |
| £90-100K | 214 |
| >100K | 650 |
|  |  |
| **Ages** | **Counts** |
| 16 | 163 |
| 17 | 130 |
| 18 | 138 |
| 19 | 140 |
| 20 | 162 |
| 21 | 145 |
| 22 | 189 |
| 23 | 166 |
| 24 | 168 |
| 25 | 221 |
| 26 | 236 |
| 27 | 285 |
| 28 | 269 |
| 29 | 289 |
| 30 | 272 |
| 31 | 269 |
| 32 | 264 |
| 33 | 246 |
| 34 | 257 |
| 35 | 254 |
| 36 | 231 |
| 37 | 250 |
| 38 | 286 |
| 39 | 310 |
| 40 | 301 |
| 41 | 278 |
| 42 | 264 |
| 43 | 256 |
| 44 | 231 |
| 45 | 309 |
| 46 | 324 |
| 47 | 290 |
| 48 | 288 |
| 49 | 300 |
| 50 | 339 |
| 51 | 298 |
| 52 | 289 |
| 53 | 310 |
| 54 | 286 |
| 55 | 308 |
| 56 | 261 |
| 57 | 252 |
| 58 | 258 |
| 59 | 247 |
| 60 | 238 |
| 61 | 173 |
| 62 | 202 |
| 63 | 192 |
| 64 | 174 |
| 65 | 191 |
| 66 | 129 |
| 67 | 117 |
| 68 | 106 |
| 69 | 84 |
| 70 | 86 |
| 71 | 59 |
| 72 | 47 |
| 73 | 60 |
| 74 | 34 |
| 75 | 30 |
| 76 | 13 |
| 77 | 20 |
| 78 | 8 |
| 79 | 11 |
| 80 | 10 |
| 81 | 3 |
| 82 | 11 |
| 83 | 2 |
| 84 | 4 |
| 85 | 1 |
| >85 | 15 |

Table S2. Profile of responses on individual IES-R items. Data are effect sizes, relative to the no breathing problem group.

| Effect size (SD units) | | | | | ANOVA |  |
| --- | --- | --- | --- | --- | --- | --- |
| No breathing  problems | Breathing problems  unassisted | Assistance  at home | Hospital/ no ventilator | Hospital + ventilator | p | IES-R Question Theme |
| 0 | 0.05 | 0.2 | 0.19 | 0.13 | 0.002 | Thinking about it when not meaning to |
| 0 | 0.02 | 0.09 | 0.04 | 0.2 | 0.2952 | Avoiding talking about it |
| 0 | 0.01 | 0.13 | 0.04 | 0.23 | 0.2021 | Waves of strong feelings about it |
| 0 | 0.04 | 0.09 | 0.04 | 0.22 | 0.0994 | Heightened arousal when not appropriate to situation |
| 0 | 0 | 0.02 | 0.13 | 0.24 | 0.2181 | Try not to think about it |
| 0 | 0.01 | 0.14 | 0.09 | 0.28 | 0.0602 | Try to remove it from memory |
| 0 | 0.02 | 0.04 | 0.14 | 0.25 | 0.1386 | External events make the person think about it |
| 0 | 0.01 | 0.07 | 0.17 | 0.32 | 0.0303 | Avoidance of situations that remind about it |
| 0 | 0.06 | 0.28 | 0.16 | 0.47 | <0.001 | More startled/nervous |
| 0 | 0.03 | 0.12 | 0.53 | 0.63 | <0.001 | Intrusive imagery |
|  |  |  |  |  |  |  |

Table S3. Pair-wise tests for comparisons of responses on different IES-R items.

| Permutation p (two tailed) vs no respiratory symptom group | | | | |
| --- | --- | --- | --- | --- |
| Breathing problems unassisted | Assistance at home | Hospital/ no ventilator | Hospital + ventilator | IES-R Question theme |
|  |  |  |  |  |
| 0.035 | 0.019 | 0.032 | 0.554 | Thinking about it when not meaning to |
| 0.451 | 0.492 | 1.289 | 0.237 | Avoiding talking about it |
| 1.49 | 0.179 | 1.289 | 0.158 | Waves of strong feelings about it |
| 0.079 | 0.412 | 1.313 | 0.168 | Heightened arousal when not appropriate to situation |
| 1.793 | 1.585 | 0.23 | 0.123 | Try not to think about it |
| 1.244 | 0.127 | 0.483 | 0.054 | Try to remove it from memory |
| 0.829 | 1.291 | 0.206 | 0.104 | External events make the person think about it |
| 1.143 | 0.751 | 0.083 | 0.02 | Avoidance of situations that remind about it |
| 0.011 | <0.001 | 0.079 | 0.001 | More startled/nervous |
| 0.379 | 0.228 | <0.001 | <0.001 | Intrusive imagery |

Copy of questions administered

| **Have you been diagnosed with a neurological or psychiatric condition?** | | | |
| --- | --- | --- | --- |
| Dementia, |  |  | depression, |
| Multiple Sclerosis, | |  | anxiety, |
| Huntington's disease, | |  | attentional deficit hyperactivity disorder, |
| Parkinson's disease, | |  | obsessive compulsive disorder, |
| Stroke, |  |  | bipolar, |
| Motor neuron disease, | |  | other psychiatric, |
| A learning disability, | |  | none of the above |
| cerebral palsy, |  |  |  |
| traumatic brain injury, | |  |  |
| other neurological, | |  |  |
| none of the above | |  |  |
|  |  |  |  |
| **Have you been diagnosed with any of the following conditions?** | | | |
| Lung conditions (e.g. asthma, emphysema or bronchitis), | | | |
| Heart disease, |  |  |  |
| Chronic kidney disease, | |  |  |
| Liver disease (e.g. hepatitis), | |  |  |
| Diabetes, |  |  |  |
| High blood pressure, | |  |  |
| Irregular heart beat (atrial fibrillation), | | |  |
| Problems with your spleen (e.g. sickle cell disease, or if you have had your spleen removed), | | | |
| A weakened immune system as the result of a condition such as HIV or AIDS, or medicines such as steroid tablets or chemotherapy., | | | |
| None of the above | |  |  |
|  |  |  |  |
| **Have you had, or suspect you have had symptoms of COVID-19?** | | | |
| Yes, currently experiencing symptoms, | | |  |
| Yes, but the symptoms have passed, | | |  |
| No |  |  |  |
|  |  |  |  |
| **Which symptoms did/do you have?,** | | |  |
| Fever, |  |  |  |
| Tiredness, |  |  |  |
| Dry cough, |  |  |  |
| Shortness of breath, | |  |  |
| Aches and pains, | |  |  |
| Sore throat, |  |  |  |
| Diarrhoea, |  |  |  |
| Nausea, |  |  |  |
| Runny nose, |  |  |  |
| Loss of smell , |  |  |  |
| Loss of taste |  |  |  |
|  |  |  |  |
| **Have you had a positive test for COVID-19?** | | |  |
| Yes, |  |  |  |
| No/don't know/awaiting test results | | |  |
|  |  |  |  |
| **Did you experience breathing difficulties?** | | Yes, No, Not sure | |
| Yes |  |  |  |
| No |  |  |  |
|  |  |  |  |
| **What happened as a result of your breathing difficulties?** | | | |
| I went to hospital and was put on a ventilator (breathing tube and mechanical assistance for breathing), | | | |
| I went to hospital, but was not put on a ventilator (see above for definition), | | | |
| I stayed at home and needed medical assistance (e.g. called 999), | | | |
| I stayed at home | |  |  |
|  |  |  |  |
| **Were you admitted to an Intensive Care Unit (ICU)?** | | | |
| Yes |  |  |  |
| No |  |  |  |
| Don't know |  |  |  |
|  |  |  |  |
| **Were you admitted to an Critical Care Unit?** | | |  |
| Yes |  |  |  |
| No |  |  |  |
| Don't know |  |  |  |
